# Dental Practice

**Published:** 1862-04

**Authors:** 


### DENTAL PRACTICE.

WE would direct the attention of our readers to the notice in the advertising sheet of the present number, headed a "Rare Chance;" and we can say, from our own knowledge, that it is a rare chance indeed.

It is a first class business in an inland city of some ten or twelve thousand inhabitants, where there is but one other office. This business has been built up and sustained by one of our first class practitioners, for fifteen years. With him, for the most of that time. we have been intimately acquainted, and know whereof we speak. The position is a good one. T.

